# Translation and Adaptation of the Adult Developmental Coordination Disorder/Dyspraxia Checklist (ADC) into Asian Uzbekistan

**DOI:** 10.3390/sports11070135

**Published:** 2023-07-17

**Authors:** Saidmamatov Orifjon, Jasurbek Jammatov, Cláudia Sousa, Rita Barros, Olga Vasconcelos, Paula Rodrigues

**Affiliations:** 1 Theory and Methodology of Physical Culture, Faculty of Physical Culture, Urgench State University, Urgench 220100, Uzbekistan; jasurjammatov@gmail.com; 2 Motor Control and Learning Laboratory, CIFI2D, Centre of Research, Education, Innovation and Intervention in Sport, Faculty of Sport, University of Porto, 4200-450 Porto, Portugal; olgav@fade.up.pt (O.V.); paula.rodrigues@ipiaget.pt (P.R.); 3 Faculty of Psychology, Education and Sports, Lusófona University, 4000-098 Porto, Portugal; p5315@ulusofona.pt; 4 RECI-Research Unit in Education and Community Intervention, Superior School of Health, Piaget Institute, Vila Nova de Gaia Portugal, 4405-678 Porto, Portugal; rita.barros@ipiaget.pt; 5 Kinesiolab, Laboratory of Human Movement Analysis, Superior School of Sports and Education, Piaget Institute, 4405-678 Vila Nova de Gaia, Portugal

**Keywords:** Adult, Developmental Coordination Disorder (DCD), motor

## Abstract

Developmental Coordination Disorder (DCD) is a neurodevelopmental condition that emerges in childhood and its symptoms continue through adulthood. The Adult Developmental Coordination Disorders/Dyspraxia Checklist was the first instrument used to screen adults with possible DCD. The psychometric characteristics of the Uzbek version of the scale were explored. An independent translation of the questionnaire from the original version into the Uzbek language was made. The sample comprised 301 Uzbekistan adults, aged between 17 and 42 years old (M = 20.66; SD = 2.26). Analyses were performed using R software (4.1.0). Descriptive analysis, exploratory factor analysis, and evidence of reliability in terms of internal consistency were assessed using the psych package (2.1.6), intraclass correlation coefficients were assessed using the irr package (0.84.1), and weighted Kappa were assessed using vcd package (1.4-10). To analyse the factor structure of the ADC scale, when applied to an Uzbekistan sample, exploratory factor analysis (EFA) was performed. In the Uzbek version, a one-factor structure was identified, and moderated psychometric properties were found, which makes it a possible alternative to the original scale when applied to adults. This Uzbek version reduces respondents’ fatigue since it is smaller than the original one. More studies are needed to confirm the cut-off scores of this new version.

## 1. Introduction

Developmental coordination disorder (DCD) is a condition that is manifested in early childhood [1], but it is well well-known that DCD often persists into adolescence and adulthood, if there is no professionally applied intervention to enable the child or young person to overcome, through a multimodal exercise programme, the diagnosed disorder. Currently, there are no explicit diagnostic criteria for adults. However, since the DSM-5 [2], adults have been mentioned. This implies that it can be used the same criteria as for children, with small adaptations of the daily activities and examples.

Studies that have considered adults with DCD are recent and scarce, revealing a negative impact of having the condition on everyday life skills [3,4,5,6,7]. They also expose that adults, like children, demonstrate a wide range of motor control (e.g., fine and gross motor skills and motor coordination), as well as poorer mental health (e.g., depression, anxiety, and stress) and physical health (e.g., obesity, cardiovascular problems, and reduced physical ability) [7,8,9,10,11]. Nevertheless, the ability of adults to develop coping mechanisms to deal with the restrictions associated with the disorder is reported and includes strategies like keyboarding instead of handwriting, using assistive applications (e.g., mobile phones and alarms), focusing on their individual strengths, and avoiding situations that reveal their motor difficulties [6].

As pointed out by Blank [12], one of the several questions and issues about the DCD to be answered and addressed for adolescents and adults concerning the diagnosis and assessment. Compared to children, where several instruments are available such as the Movement Assessment Battery for Children, second edition (MABC-2) [13], the most relevant between them, or the Bruininks–Oseretsky Motor Proficiency Test, second edition (BOT-2) [14], for adults; however, there are few tools available for the diagnosis and assessment of DCD. Aiming to map the assessments used for this population, a scoping review was made [15]. The results revealed that the Adult Developmental Coordination/Dyspraxia Checklist (ADC) [16] has been the most widely used instrument, together with other self-report screening questionnaires such as Adolescents and Adults Coordination Questionnaire [17] and Functional Difficulties Questionnaire [18]. However, it is recommended that further work with these instruments is conducted to establish their psychometric properties [13].

Recognising that the study of DCD in adults is scarce in international investigations, the authors also emphasise the fact that this line of research has not devoted the analysis that the topic deserves when it comes to studying adults from different cultures. Therefore, in order to allow comparability in the field of DCD research among adults from different cultures, the authors have developed a process of cultural adaptation and validation of the ADC in different countries. This procedure will allow analyses leading to answers to long-standing questions such as what is the percentage of adults with DCD? what is the percentage of adults with DCD relative to the percentages of children and young people?; whether these percentages differ between sexes; whether these percentages have decreased in adults who have frequently participated in physical activities and sports. It is already in development by the authors for the cultural adaptation of the ADC for Portugal. In view of these considerations, the authors aim to embrace a study involving a preliminary translation and validation of the ADC for the adult Uzbek population. Moreover, until now, some researchers have studied the coordination problems of preschool children in Uzbekistan, but the coordination problems of adults have not been studied so far. This research directly serves to expand the scope of scientific research conducted among children with DCD in this direction in the territory of Uzbekistan. The main aim of our study is to investigate the psychometric properties of the Uzbek version of the scale, as well as to determine if it is a valid instrument to be used not only in a clinical setting but also in terms of scientific research.

## 2. Materials and Methods

### 2.1. Participants

The sample was defined by convenience, according to accessibility, and comprises 301 Uzbekistan university students (59.1% males and 40.9% females), aged between 17 and 42 years old (20.66 ± 2.26). Inclusion criteria for this were Uzbek natives and at least 17 years old. Exclusion criteria were difficulties in written comprehension and individuals with other severe psychopathologies.

The questionnaire was applied a second time, after one week, to the same sample.

### 2.2. Instrument

The ADC is a self-report questionnaire that is practical, simple to complete, and brief (it takes 15 to 20 min to complete). It provides information on the adult’s ability to act in different contexts such as the home, academic, and social environments. It consists of 40 items that relate to space and time organisation while performing daily life activities and self-care skills, common academic and vocational activities, hobbies, and social participation capacity.

The questionnaire’s structure includes three subscales: subscale A (10 items), related to difficulties experienced during childhood; subscale B (20 items), focused on the influence of DCD on the individuals’ perception of their performance as adults; subscale C (10 items), related to the current feelings that individuals have about their performance but as reflected upon by others.

A 4-point Likert scale (1—“never” to 4—“always”) was used for item’s evaluation. Lower scores correspond to better performance.

The authors of the original scale analysed the instrument’s validity including content validity. For that purpose, the original 40-item questionnaire was, initially, reviewed by three expert consultants and five clinicians. Three adults that described themselves as clumsy answered the questionnaire and, after completing it, discussed each item in terms of intelligibility and ease of response. Afterward, the questionnaire was applied to a sample of 107 students, between 17 and 42 years, of whom 49 were included in the DCD group, and 58 were included in the control group.

Internal consistency reliability was assessed using Cronbach’s alpha (α = 0.95 for the overall scale, α = 0.91 for subscale A, α = 0.87 for subscale B, and α = 0.90 for subscale C) and Pearson correlations were used to analyse the correlations between each one of the ADC items and the ADC final score (r = 0.44–0.79, *p* < 0.001).

Results showed that the ADC (and each subscale) were able to distinguish the DCD group from the control group. Kirby et al. [17] set cut-off scores of 80 and above as “probable” difficulties and scores of 90 and above as “likely” difficulties. However, Kirby et al. [19] revised these cut-off scores. Indeed, the authors considered the items measured on a scale coded from 0 to 3 and suggested cut-off scores of 56 and above as “at risk of DCD” and scores of 65 and above as “with probable DCD”, which, on a scale coded from 1 to 4 (as the one considered in the present study), correspond to cut-off scores of 75 and 87, respectively.

#### Procedures

In this present study, several steps were taken to adapt the ADC scale to the Uzbekistan context. Prior to its administration to a sample of Uzbekistan adults, its items were translated into the Uzbek language. Five Uzbek natives, including four that were English professors from Urgench State University, and one from the Faculty of Physical Culture, at the same University, independently translated the questionnaire from its original version into Uzbek. The lexical and grammatical equivalence, as well as content validity, were verified by a group of experts trained in Sports and English. Any doubts resulting from this process were discussed in three working sessions among them until a consensus solution was found. Subsequently, the assessment of the initial translation was based on a retroversion that was carried out by an English teacher with skills in the field of translation. This retroversion was compared with the original English instrument. As no significant discrepancies were detected, a version of the translated instrument was found.

Table 1 shows the 40 items of the ADC questionnaire, with corresponding Uzbek translations and original subscales.

The ADC questionnaire together with sociodemographic questions was applied to a sample of 301 Uzbekistan university students. The survey was conducted on the google forms platform and the survey link was given to the students through the telegram social network. All of them were informed about the anonymous and voluntary nature of the study and gave informed consent to participate in the research. All respondents accepted to respond a second time and were contacted one week later. This study was approved by the Ethics Commission of Urgench State University.

### 2.3. Data Analysis

R software (version 4.1.0) was used to perform the analyses. Psych package (2.1.6), was used to carry out descriptive analysis and exploratory factor analysis. Irr package (0.84.1) was used to assess the evidence of reliability in terms of internal consistency via intraclass correlation coefficients. Weighted Kappa was calculated using the vcd package (1.4-10).

Two of the items, namely items 25 and 32 (see Table 1) related to driving, had several missing answers, due to individuals that do not have a driving license. The missing answers to these items were coded with 0, and 0 was identified as a missing value. Since the questionnaire is also applicable to individuals that do not drive, pairwise deletion procedures were applied to consider the responses that these individuals gave to the other items.

Exploratory factor analysis (EFA) was performed to analyse the factor structure of the ADC scale when applied to the Uzbek sample. The Bartlett sphericity test and Kaiser–Meyer–Olkin index (KMO) statistic were used to determine if the data was adequate for performing EFA. A significant sphericity test and KMO ≥ 0.70 were used as guidelines for data adequacy [20] EFA was then performed using principal axis factoring (PA). PA is a least-squares estimation method that makes no assumptions about the data distribution [21] and least-squares estimation methods increase the likelihood of recovering all major common factors [22]. Since, based on Meachon et al.’s [23] results, it was assumed that factors would be correlated and oblimin rotation was considered. A visual inspection of the scree plot (which, according to Pestana and Gageiro [24], should be used when the number of items is higher than 30) and Horn’s method of parallel analysis [25] were used to decide the number of factors to retain, and the 0.40/0.30/0.20 rule for the factor loading cut-off was considered [26]. According to this rule, satisfactory variables should load onto their primary factor above 0.40, alternative factors below 0.30, and the difference between their primary and alternative factor loadings should be 0.20.

For EFA with ordinal items, Spearman correlations were considered according to the guidelines of Pestana and Gageiro [24], since the ordinal items had only four response categories and were not symmetrical.

Reliability was assessed by the internal consistency of the factors using Cronbach’s alpha. Values of 0.70 or higher were deemed sufficient [27]. Test–retest reliability of each item, of subscales, and of the total score were also assessed. Measurements were repeated two times for everyone. Intraclass correlation coefficients (ICC), using single-measurement, absolute-agreement, and 2-way mixed effects models [28], were then used to evaluate test–retest reliability of the subscales and of the total score. ICC values less than 0.50, between 0.50 and 0.75, between 0.75 and 0.90, and greater than 0.90 are indicative of poor, moderate, good, and excellent reliability [28]. Since the items are ordinal, the test–retest reliability of each item was evaluated by the Kappa coefficient. Quadratic weights were used since its interpretation is similar to the ICC [29].

The strength of agreement is considered poor, slight, fair, moderate, substantial, and almost perfect if kappa values are <0.00, between 0.00 and 0.20, between 0.21 and 0.40, between 0.41 and 0.60, between 0.61 and 0.80, or between 0.80 and 1.00, respectively [30]. ICC and Kappa, point estimates, and 95% confidence intervals are presented.

Similarly to what was conducted in Kirby et al. [16] correlations between the ADC factors were calculated. Spearman correlations between each one of the ADC items and the ADC final score are also presented.

Correlations between both total scores and Spearman correlations between age and the total scores of both scales are also presented (Spearman correlations were taken into account, considering the asymmetric nature of the variables). T-tests were also applied to analyse the differences between genders for both total scores. The identification of each individual as “without DCD”, “at risk of DCD”, and “with probable DCD”, when using the total score of both scales, was compared.

## 3. Results

Descriptive statistics of all the 40 items of the original ADC scale, when applied to the Uzbekistan sample, are presented in Table 2. As can be seen, all four response categories were used (Min = 1, Max = 4), except for item 29 (“Do you avoid team games/sports?”; Min = 1, Max = 3) and all items present medians and means that are between one and approximately two, which means that, in general, our sample never had difficulties performing the activities or only had difficulties sometimes.

The correlations between the three original subscales were moderate (r = 0.54 between subscale A and subscale B; r = 0.55 between subscale A and subscale C; r = 0.65 between subscales B and C) and Cronbach’s alphas indicated adequate internal consistency reliability of the overall scale (α = 0.90) and subscales B and C (α = 0.76 for subscale B; α = 0.85 for subscale C), but the internal consistency reliability of subscale A was not adequate (α = 0.65).

Results of the Bartlett sphericity test (χ^2^ (780) = 3188.232; *p* < 0.001), as well as KMO = 0.87, suggested that this data was adequate for performing exploratory factor analysis. After performing Horn’s parallel analysis method and visualising the Scree Plot, the analyses led to the extraction of only one factor and, after applying the 0.40-0.30-0.20 rule for the factor loading cut-off, 11 items were eliminated (items 1, 3, 4, 6, 8, 10, 12, 24, 28, 29, and 31). Table 2 shows the loadings of each of the remaining 29 items. As can be seen, all those remaining items had adequate factor loadings (of 0.40 or higher) and, following rotation, the extracted factor accounted for 23% of the total variance. The item–total correlations were all statistically significant (*p* < 0.001) and ranged between 0.37 (item 11) and 0.58 (items 9 and 40) (see Table 2). Cronbach’s alpha indicated a strong internal consistency reliability of the revised scale (α = 0.89).

The test–retest reliability of the original scale was moderate for subscale A (ICC = 0.54, 95% CI = 0.46–0.62, *p* < 0.001), poor for subscale B (ICC = 0.46, 95% CI = 0.36–0.54, *p* < 0.001), poor for subscale C (ICC = 0.49, 95% CI = 0.40–0.58, *p* < 0.001), and moderate for the total score (ICC = 0.54, 95% CI = 0.45–0.61, *p* < 0.001). After the 11 items were eliminated, test–retest reliability of the new factor structure was moderate (ICC = 0.53, 95% CI = 0.44–0.61, *p* < 0.001).

The Kappa coefficient estimates with their 95% confidence intervals, for each item, can be seen in Table 3.

According to the Kappa values of Table 3, the strength of the agreement is considered as fair or moderate, for almost all items. The only exception is item 17 (“Is copying things down without mistakes difficult?”) since the obtained Kappa value indicates a slight agreement.

The total score of both scales (original and adapted one) are almost perfectly correlated (r = 0.97, *p* < 0.001), and correlations between age and the total score of both scales were not statistically significant (original scale: r = −0.04, *p* = 0.460; adapted scale: r = −0.01, *p* = 0.859). There were no differences between genders when considering the total score of the original scale [t (299) = 1.11, *p* = 0.270)]. However, when considering the total score of the adapted scale, males scored higher (M = 41.83; DP = 9.00) than females (M = 39.67; DP = 8.10) [(t (299) = 2.14, *p* = 0.034)].

Since the authors of the original scale considered that cut-off scores of 75 and above were indicative of “at risk of DCD” and scores of 87 and above were indicative of “with probable DCD”. Since in our proposal we only consider 29 items, the considered cut-off scores were 55 and above for “at risk of DCD” and 64 and above for “with probable DCD”. When comparing the allocation of each individual to one of the groups related to DCD (“without DCD”, at risk of DCD”, and “with probable DCD”) after using both total scores, it was verified that 277 of the 301 individuals (92.03%) were equally allocated. Analysing the remaining 24 individuals, the revised scale identified two of them as “with probable DCD” while the original scale identified them as “at risk of DCD” and there were 22 individuals that the original scale identified as not having DCD, but our scale identified as “at risk of DCD”.

## 4. Discussion

When performing EFA on an Uzbekistan sample, the original structure of the 40-item ADC scale did not hold. Instead, only one factor was extracted and eleven of the original items had to be removed, namely, items 1, 3, 4, 6, 8, 10, 12, 24, 28, 29, and 31. Items 8, 28, and 31 were also removed in Meachon et al. [23]. We are in line with the interpretation of these authors concerning social interactions for items 28 (desire to spend time alone) and 31 (go dancing at a club) on which it highlighted the applicability to the secondary symptoms of DCD based on general social preferences instead of the applicability to first symptoms of DCD, concerning fine motor, gross motor, and executive function subscales. Decreased peer interaction that frequently characterises individuals with DCD may result from their low perceptions of athletic and scholastic competence [31].

The same can be applied to item 29 because team sports, although involving gross motor skills, they require also social interactions. It is possible that both factors may lead to social challenges, resulting from the possible lower perceived social support and negative self-concept that has been documented in adults with DCD [7]. Nevertheless, it is important to consider respondents’ social preferences independent from DCD to better interpret and contextualise the results obtained.

Cultural reasons can justify the option to remove some items such as items 3 and 12. Riding a bike is not a regular activity in Uzbekistan during wintertime and eating with hands is a common practice. Playing a musical instrument is addressed in item 8. Its removal can be explained both by cultural factors and financial issues that do not offer the opportunity to play it. Also, the elimination of item 4 may be attached to Governmental legal dispositions at the school level. The reduced duration and frequency of attendance (once per week, 1h each class, and a high number of children in each class) may contribute to a very reduced motor engagement time. Consequently, the difficulties are probably due to a lack of opportunities for normal motor development and not to a neurodevelopmental problem.

Developmental factors can also explain the elimination of items 1 and 6. Difficulties with self-care tasks and writing as a child may be age-related, with some skills, like brushing one’s teeth and clothing oneself, seem to improve for many as they can be automated as the external environment becomes more stable and predictable [19].

When comparing the total scores of the original scale and the one adapted to the Uzbekistan context, they were almost, perfectly correlated, and when comparing both total scores with age, no statistically significant correlations were found.

Regarding gender, no statistically significant differences were found when considering the original scale total score, which is in clear disagreement with a recent study by Cleaton et al. [32]. However, the total score of the revised scale is in line with this study, since differences between genders were found. However, further studies are needed to deepen our knowledge of the relationship between gender and this disorder.

This instrument and its cut-offs allow a previous identification of pathologic situations that could be oriented to clinical services for an accurate clinical diagnostic. It works as a screening tool for professionals working in the field.

When identifying individuals in one of the groups “without DCD”, “at risk of DCD”, and “with probable DCD”, only 24 of the 301 analysed cases differed when using both scales. Also, of the 24 cases that both scales allocated differently in the groups “without DCD”, “at risk of DCD”, and “with probable DCD”, the new scale never identified an individual as not having DCD, which seems to indicate that a higher accuracy of the Uzbek version.

Concerning the reliability of both scales, measured in terms of internal consistency, both overall scales presented Cronbach’s alphas indicating strong internal consistency reliability (similarly to [15,22]), but the internal consistency reliability of subscale A form the original scale was not adequate. Furthermore, the test–retest reliability of subscales B and C from the original scale was poor.

In line with Kirby et al. [16] in this adaptation, correlations between each of the 29 retained items and the final score of the new version of the ADC are quite similar.

The analysis of the factorial structure of the ADC scale, though EFA, when applied to an Uzbekistan sample, is a novelty of the present study. To our knowledge, there is no other study focused on adaptation of this scale to an Asian population.

One of the advantages of this scale adaptation was the use of test–retest reliability analysis.

The size of this Uzbek version reduces the respondent’s fatigue since it is smaller than the original one. It also presents moderated psychometric properties, which makes it a possible alternative to the original scale, when applied to Uzbekistan adults. However, the choice of a convenience sample that includes only university students is a limitation of our study and further studies with a wider age range of adults is recommended.

The total variance explained was below our expectations and before recommending this scale for clinical and research use it requires deeper analysis such as presented by Meachon [23] that can include confirmation of the cut-off scores of this new scale, as was already proposed by Kirby et al. [16] for the original scale.

In conclusion, the Uzbek version of this scale is unidimensional and smaller one, when comparing to the original version. However, its psychometric properties are preserved, which leads us to recommend its use in several contexts including the educational ones. Nevertheless, although the questionnaire seems to be adequate, it is considered that there is an opportunity for improvement, namely, to see if it discriminates correctly between those with DCD and those without DCD. Studies to confirm the cut-off scores of this new scale are suggested, as was already proposed by Kirby et al. [16] for the original scale. It is encouraging that DCD in adults has recently attracted more global notice and that international clinical practice guidelines were published [12]. We believe it is important that more psychological researchers and practitioners around the world pay attention to this growth and, if possible, incorporate this kind of screening test into their work. This is crucial for enhancing the symptom profiles, prevalence projections, differential diagnoses, and treatment effectiveness of DCD symptoms across all age groups. As mentioned by some authors [32,33,34], instrument validation assumes particular importance in tasks of evaluation, diagnosis and monitoring of psychological intervention. The early screening of adults with DCD may allow the establishment of services that include prevention and intervention programs that contribute to the use of coping strategies to enhance function and participation in their real-life context. It is also important to raise awareness of DCD patients, their families, and other stakeholders about the challenges that adults with DCD experience.

## Figures and Tables

**Table 1:** ADC questionnaire, with corresponding Uzbek translation and original subscales.

Item
***Subscale A (as a child):***
1. Do you have difficulties with self-care tasks such as tying shoelaces, fastening buttons, and zips? (Oyoq kiyimlarni bog‘lash, tugmalarni mahkamlash yoki zamoklarni ochish va yopish kabi o‘zingiz bajarishingiz kerak bo‘lgan ishlarni bajarishda qiynalganmisiz?)
2. Do you have difficulty eating without getting dirty? (Kiyimlaringizni iflos qilmasdan ovqatlanishga qiynalganmisiz?)
3. Did you have difficulty learning to ride a bike compared to your peers? (Sizda yoshingizdagi boshqa bolalarga qaraganda velosiped haydashni o‘rganishda qiynchiliklar bo‘lganmi?)
4. Do you have difficulties with playing team games such as football, volleyball, catching, or throwing balls accurately? (Sizda futbol, voleybol, to‘pni ushlab olish yoki uloqtirish kabi jamoaviy sport turlarini o‘ynashda qiyinchiliklar bo‘lgami?)
5. Do you have difficulty writing neatly (so others can read it)? (Sizda tushunarli qilib yozishda qiyinchilik bo‘lganmi (boshqalar o‘qib tushunishi uchun)?)
6. Do you have difficulty writing as fast as your peers? (Tengdoshlaringiz kabi tez yozishda qiynalganmisiz?)
7. Do you bump into objects or people or trip over things more than others? (Boshqalarga nisbatan buyum yoki insonlarga ko‘proq qoqilib yoki urilib ketganmisiz?)
8. Do you have difficulty playing a musical instrument (e.g., violin or recorder)? (Musiqiy asboblarni (skripka, nay kabi) chalishda qiyinchiliklar bo‘lganmi?)
9. Do you have difficulties with organising/finding your things in your room? (Xonangizdagi narsalarni tartibga solish/topishda qiyinchilik bo‘lganmi?)
10. Have others commented on your lack of co-ordination or call you clumsy? (Boshqalar sizning e’tiborsizligingiz to‘g‘risida fikr bildirgan yoki sizni noʻnoq deb atishganmi?)
***Subscale B (current symptoms):***
11. Are self-care tasks, such as shaving or make-up, difficult for you? (Soqol olish yoki pardoz qilish kabi mustaqil bajariluvchi vazifalarda qiyinchilik mavjudmi?)
12. Is eating with knife and fork/spoon difficult? (Pichoq va vilkalar/qoshiq bilan ovqatlanishda?)
13. Are hobbies that require good co-ordination difficult? (Yaxshi koordinatsiyani talab qiluvchi sevimli mashg‘ulotlarni bajarishda?)
14. Is writing neatly when having to write fast difficult? (Tez yozishga to‘g‘ri kelganda chiroyli yozishda?)
15. Is writing as fast as your peers difficult? (Tengdoshlaringiz kabi tez yozishda?)
16. Is reading your own writing difficult? (O‘z yozuvingizni o‘qishda?)
17. Is copying things down without mistakes difficult? (Matnlarni xato qilmasdan ko‘chirib yozishda?)
18. Is organising/finding your things in your room difficult? (Xonangizdagi narsalarni tartibga solishda/topishda?)
19. Is finding your way around new buildings or places difficult? (Siz uchun yangi boʻlgan joylarda yoki binolarda oʻzingiz yo‘l topa olasizmi?)
20. Have others called you disorganized? (Boshqalar sizni tartibsiz deb atashganmi?)
***Subscale C (current symptoms manifested by others):***
21. Do you have difficulties with sitting still or not appearing fidgety? (Sizda jim o‘tirish yoki xotirjamlikni saqlashda muommo bormi?)
22. Do you lose or leave behind possessions? (Shaxsiy narsalaringizni tez-tez yoʻqotasizmi yoki unutib qoldirasizmi?)
23. Would you say that you bump into things, spill or break things? (Siz o‘zingizni biror narsalarga urilib ketish, narsalarni to‘kish yoki tushirishga moyil deb ayta olasizmi?)
24. Are you slower than others getting up in the morning and getting to work or college? (Ertalab turib, ishga yoki kollej ga borishda boshqalarga qaraganda sekinroq harakatlanasizmi?)
25. Did it take you longer than others to learn to drive? (Mashina haydashni o‘rganishingiz uchun boshqalarga qaraganda ko‘proq vaqt kerak bo‘ldimi?)
26. Do others find it difficult to read your writing? (Boshqalar sizning husnixatingizni o‘qishni qiyin deb bilishadimi?)
27. Do you avoid hobbies that require good co-ordination? (Yaxshi koordinatsiyani talab qiladigan sevimli mashg‘ulotlaringizdan qochasizmi?)
28. Do you choose to spend leisure time more on your own than with others? (Bo‘sh vaqtingizni boshqalar bilan oʻtkazishdan ko‘ra ko‘proq o‘zingiz o‘tkazishni afzal ko‘rasizmi?)
29. Do you avoid team games/sports? (Sport oʻyinlari/jamoaviy o‘yinlaridan o‘zingizni olib qochganmisiz?)
30. If you do a sport, is it more likely to be on your own, e.g., going to a gym, than with others? (Agar siz sport bilan shug‘ullansangiz, boshqalar bilan emas, balki yolg‘iz qolish ehtimoli ko‘proqmi (masalan, sport zaliga borish)?)
31. Did you tend to in your teens/twenties or currently avoid going to clubs/dancing? (O‘smirlik davrida/20 yoshda siz raqs to‘garagi/sport to‘garagiga (klublari) borishdan oʻzingizni olib qochganmisiz?)
32. If you are a driver, do you have difficulty parking a car? (Agar siz mashina haydasangiz, avtomobil to‘xtash joyiga mashinangizni qo‘yishda qiynalasizmi?)
33. Do you have difficulty preparing a meal from scratch? (Ovqat tayyorlash/pishirish paytida qo‘lingizni kesib olish/tirnab olish kabi muammoga duch kelyapsizmi?)
34. Do you have difficulty packing a suitcase to go away? (Sayohat uchun chamadonni yig‘ishda muammoga duch kelasizmi?)
35. Do you have difficulty folding clothes to put them away neatly? (Kiyimlaringizni ozoda saqlash uchun ularni taxlab qo‘yish siz uchun qiyinmi?)
36. Do you have difficulty managing money? (Pulingizni boshqarishda qiynalasizmi?)
37. Do you have difficulties with performing two things at the same time (e.g., driving and listening)? (Bir vaqtning o‘zida ikkita vazifani bajarishda qiynalasizmi (masalan, mashina haydash va eshitish yoki uyali telefondagi xabarni o‘qish)?)
38. Do you have difficulties with distance estimation (e.g., with regard to parking, passing through objects)? (Masofalarni hisoblashda muammoga duch kelasizmi (masalan, avtomabil to‘xtash joylarida, ob’ektlar orasidan o‘tishda)?)
39. Do you have difficulty planning ahead? (Oldindan rejalar tuzishda muammoga duch kelasizmi?)
40. Do you feel you are losing attention in certain situations? (Muayyan vaziyatlarda sizda konsentratsiya yo‘qolayotganini his qilasizmi?)

**Table 2:** Descriptive Statistics of the 40 items of the ADC, factor loadings from the initial EFA with a Pakistani sample and factor loadings, communalities, and item-total correlations of the 29 items retained after EFA.

Item	*n*	*Min*	*Max*	*Me*	*M*	*SD*	Factor (Pattern) Loadings		*h* ^2^	Item-Total Correlations
							Initial EFA (All Items)	Reduced Model (29 Items)		
Item 1	301	1	4	1	1.44	0.66	0.38	-	-	-
Item 2	301	1	4	1	1.39	0.56	0.43	0.41	0.17	0.42
Item 3	301	1	4	1	1.41	0.74	0.24	-	-	-
Item 4	301	1	4	1	1.47	0.59	0.36	-	-	-
Item 5	301	1	4	1	1.5	0.65	0.4	0.4	0.16	0.48
Item 6	301	1	4	1	1.42	0.56	0.36	-	-	-
Item 7	301	1	4	2	1.66	0.6	0.41	0.41	0.17	0.43
Item 8	301	1	4	2	2.16	1.14	0.12	-	-	-
Item 9	301	1	4	1	1.56	0.65	0.54	0.54	0.29	0.58
Item 10	301	1	4	1	1.32	0.53	0.38	-	-	-
Item 11	301	1	4	1	1.24	0.47	0.43	0.42	0.18	0.37
Item 12	301	1	4	1	1.24	0.6	0.31	-	-	-
Item 13	301	1	4	1	1.31	0.55	0.41	0.4	0.16	0.41
Item 14	301	1	4	2	1.82	0.74	0.42	0.42	0.17	0.49
Item 15	301	1	4	1	1.53	0.71	0.44	0.43	0.19	0.5
Item 16	301	1	4	1	1.3	0.64	0.45	0.46	0.21	0.47
Item 17	301	1	4	1	1.49	0.65	0.49	0.5	0.25	0.55
Item 18	301	1	4	1	1.43	0.64	0.53	0.54	0.3	0.55
Item 19	301	1	4	2	1.62	0.75	0.53	0.53	0.28	0.55
Item 20	301	1	4	1	1.31	0.54	0.56	0.56	0.32	0.53
Item 21	301	1	4	1	1.31	0.59	0.48	0.5	0.25	0.47
Item 22	301	1	4	2	1.73	0.62	0.55	0.55	0.3	0.56
Item 23	301	1	4	1	1.29	0.5	0.48	0.48	0.23	0.46
Item 24	301	1	4	1	1.34	0.58	0.36	-	-	-
Item 25	178	1	4	1	1.27	0.52	0.46	0.47	0.22	0.47
Item 26	301	1	4	1	1.54	0.67	0.42	0.42	0.17	0.5
Item 27	301	1	4	1	1.27	0.52	0.48	0.47	0.22	0.43
Item 28	301	1	4	2	1.87	0.81	0.27	-	-	-
Item 29	301	1	3	1	1.29	0.51	0.3	-	-	-
Item 30	301	1	4	1	1.55	0.73	0.44	0.42	0.18	0.46
Item 31	301	1	4	1	1.7	1	0.27	-	-	-
Item 32	178	1	4	1	1.44	0.75	0.55	0.57	0.32	0.57
Item 33	301	1	4	2	1.64	0.65	0.46	0.45	0.21	0.45
Item 34	301	1	4	1	1.31	0.55	0.59	0.6	0.36	0.54
Item 35	301	1	4	1	1.22	0.5	0.42	0.42	0.18	0.42
Item 36	301	1	4	1	1.51	0.75	0.49	0.49	0.24	0.47
Item 37	301	1	4	1	1.42	0.67	0.41	0.41	0.17	0.42
Item 38	301	1	4	1	1.43	0.59	0.55	0.56	0.31	0.52
Item 39	301	1	4	1	1.47	0.63	0.52	0.53	0.28	0.53
Item 40	301	1	4	1	1.5	0.6	0.56	0.57	0.32	0.58
% variance explained							20	23		

*Note*: *h*^2^ = communalities.

**Table 3:** Kappa coefficients with quadratic weights and the corresponding 95% confidence intervals for each of the 40 items in the ADC scale.

Item	*n*	Kappa Coefficient with Quadratic Weights (95% CI)
Item 1	301	0.27 *** (0.11–0.43)
Item 2	301	0.36 *** (0.21–0.50)
Item 3	301	0.54 *** (0.41–0.68)
Item 4	301	0.37 *** (0.27–0.48)
Item 5	301	0.41 *** (0.28–0.54)
Item 6	301	0.36 *** (0.26–0.46)
Item 7	301	0.29 *** (0.17–0.40)
Item 8	301	0.28 *** (0.16–0.39)
Item 9	301	0.33 *** (0.20–0.46)
Item 10	301	0.30 *** (0.18–0.42)
Item 11	301	0.30 *** (0.17–0.43)
Item 12	301	0.31 *** (0.13–0.50)
Item 13	301	0.38 *** (0.25–0.52)
Item 14	301	0.25 *** (0.12–0.37)
Item 15	301	0.26 *** (0.14–0.38)
Item 16	301	0.31 *** (0.15–0.46)
Item 17	301	0.17 ** (0.06–0.29)
Item 18	301	0.39 *** (0.27–0.52)
Item 19	301	0.29 *** (0.16–0.41)
Item 20	301	0.37 *** (0.26–0.49)
Item 21	301	0.30 *** (0.15–0.45)
Item 22	301	0.27 *** (0.14–0.40)
Item 23	301	0.33 *** (0.17–0.48)
Item 24	301	0.29 *** (0.15–0.43)
Item 25	178	0.21 ** (0.04–0.37)
Item 26	301	0.38 *** (0.24–0.51)
Item 27	301	0.29 *** (0.18–0.40)
Item 28	301	0.28 *** (0.17–0.39)
Item 29	301	0.34 *** (0.21–0.46)
Item 30	301	0.26 *** (0.14–0.37)
Item 31	301	0.36 *** (0.24–0.48)
Item 32	178	0.42 *** (0.22–0.62)
Item 33	301	0.28 *** (0.15–0.41)
Item 34	301	0.29 *** (0.14–0.44)
Item 35	301	0.23 *** (0.11–0.35)
Item 36	301	0.53 *** (0.40–0.65)
Item 37	301	0.28 *** (0.14–0.41)
Item 38	301	0.34 *** (0.20–0.48)
Item 39	301	0.35 *** (0.23–0.47)
Item 40	301	0.33 *** (0.20–0.47)

*Note:* *** *p* < 0.001, ** *p* < 0.01.
